# Radiopharmaceuticals for Skeletal Muscle PET Imaging

**DOI:** 10.3390/ijms25094860

**Published:** 2024-04-29

**Authors:** Joo Yeon Park, Sun Mi Park, Tae Sup Lee, Seo Young Kang, Ji-Young Kim, Hai-Jeon Yoon, Bom Sahn Kim, Byung Seok Moon

**Affiliations:** 1 Department of Nuclear Medicine, Ewha Womans University College of Medicine, Seoul 07804, Republic of Korea; jooy259@naver.com (J.Y.P.); psm9728@ewha.ac.kr (S.M.P.); eironn02@gmail.com (S.Y.K.); gotothekorea@gmail.com (J.-Y.K.); haijeon.yoon@gmail.com (H.-J.Y.); 2 Division of RI Applications, Korea Institute Radiological and Medical Sciences, Seoul 01812, Republic of Korea; nobelcow@kirams.re.kr

**Keywords:** positron emission tomography, radiopharmaceutical, skeletal muscle atrophy

## Abstract

The skeletal muscles account for approximately 40% of the body weight and are crucial in movement, nutrient absorption, and energy metabolism. Muscle loss and decline in function cause a decrease in the quality of life of patients and the elderly, leading to complications that require early diagnosis. Positron emission tomography/computed tomography (PET/CT) offers non-invasive, high-resolution visualization of tissues. It has emerged as a promising alternative to invasive diagnostic methods and is attracting attention as a tool for assessing muscle function and imaging muscle diseases. Effective imaging of muscle function and pathology relies on appropriate radiopharmaceuticals that target key aspects of muscle metabolism, such as glucose uptake, adenosine triphosphate (ATP) production, and the oxidation of fat and carbohydrates. In this review, we describe how [^18^F]fluoro-2-deoxy-D-glucose ([^18^F]FDG), [^18^F]fluorocholine ([^18^F]FCH), [^11^C]acetate, and [^15^O]water ([^15^O]H_2_O) are suitable radiopharmaceuticals for diagnostic imaging of skeletal muscles.

## 1. Introduction

The skeletal muscles constitute approximately 40% of a healthy individual’s body weight and are heterogeneous tissues composed of various types of muscle fibers [1,2]. Functionally, they are essential for facilitating movement, supporting body structure, enabling nutrient absorption, promoting blood circulation, regulating temperature, and contributing to energy metabolism [1,2,3]. The maintenance of skeletal muscle mass is intricately regulated by the balance between protein synthesis and breakdown reactions [1,4].

Muscle atrophy, characterized by the loss of mass and function in skeletal muscles resulting from an imbalance between protein synthesis and breakdown, encompasses conditions such as muscle malnutrition, muscle-wasting syndrome, and cachexia [4,5,6]. Diverse causes of muscle atrophy include insufficient physical activity, aging, disease, excessive stress, hormonal changes, and congenital factors [4,7,8,9,10,11,12]. In contemporary medical discourse, muscle atrophy is defined as a pathological degenerative condition that negatively affects the quality of life and contributes to the mortality and morbidity of various diseases [4,13].

In contrast to muscle atrophy, muscle hypertrophy involves increased muscle size [4]. Muscle hypertrophy is considered the primary treatment for muscle atrophy; however, it also interests athletes in various sports, especially people participating in bodybuilding. Muscle hypertrophy can be induced through appropriate exercise, rest, or nutritional supplementation. Exercises such as resistance training with a suitable intensity lead to minor cellular damage and protein breakdown in muscle cells. During the subsequent rest period, factors such as the mammalian target of rapamycin (mTOR) promote protein synthesis facilitated by proteins such as the ribosomal protein S6 kinase beta-1 (S6K1) [14,15,16].

The diagnosis of diseases related to skeletal muscle involves various methods for measuring muscle quantity. Techniques such as dual-energy X-ray absorptiometry (DXA), bioelectrical impedance analysis (BIA), ultrasound, electromyography, computed tomography (CT), and magnetic resonance imaging (MRI) enable examination of the overall tissue condition through anatomical imaging, localized information provision, or muscle mass insights via invasive approaches [9,15,17,18,19,20]. However, diagnosing these conditions may pose challenges in cases with no evident anatomical or pronounced neurological abnormalities.

Positron emission tomography (PET), a functional imaging modality, is a promising approach for assessing metabolic activity in the human body [21]. By utilizing positron-emitting radionuclides, PET captures metabolic activities within a patient’s body and converts them into images [22]. PET’s operational principle involves the administration of a radiopharmaceutical to a patient, where the emitted positrons undergo annihilation upon collision with electrons, generating gamma photons. PET scanners detect these photons, track positron emissions, and produce images to effectively evaluate physiological aspects such as glucose and lipid metabolism. PET is widely used in the medical field and provides high accuracy with minimal radiation exposure [21,23,24]. Radiopharmaceuticals used in PET imaging enable non-invasive, real-time visualization of biochemical processes at the cellular and molecular levels within living organisms, confirming the specific and selective binding of the target. Typically, these radiopharmaceuticals include positron-emitting radionuclides with relatively short half-lives, such as fluorine-18, carbon-11, nitrogen-13, and oxygen-15, which are produced by cyclotrons for PET imaging [21,25].

This review aims to explore the potential of PET imaging in diagnosing muscle atrophy or muscle hypertrophy and discusses the available radiopharmaceuticals and their applications.

## 2. Radiopharmaceuticals for PET Imaging of Skeletal Muscle

The literature search on PubMed was performed by two authors (J.Y.P. and S.M.P.) on 13 December 2023 to find case studies and articles relevant to the present objective. The search utilized the keywords “positron emission tomography” and “skeletal muscle imaging” without restrictions on publication date. The inclusion criteria comprised case studies and articles related to (i) preclinical and clinical studies and (ii) studies confirming muscle activation/deactivation or changes using radiopharmaceuticals. Exclusion criteria encompassed (i) reviews, meta-analyses, and conference abstracts; (ii) non-English publications; (iii) duplicated articles; (iv) inaccessible articles; and (v) articles primarily unrelated to the research objectives. Several radiopharmaceuticals have been proposed for skeletal muscle PET imaging, and various studies have been conducted on this subject (Table 1).

### 2.1. [^18^F]Fluoro-2-Deoxy-D-Glucose ([^18^F]FDG)

[^18^F]FDG uptake serves as an indicator of glucose absorption. [^18^F]FDG PET imaging has evolved into a standard diagnostic tool for the detection of various cancers. This method exploits the Warburg effect, in which cancer cells and tissues exhibit higher glucose absorption than normal cells. As [^18^F]FDG is transported through the bloodstream, the cancer cells rapidly absorb glucose. During the metabolic process, [^18^F]FDG undergoes decay, emitting radiation detected by a PET scanner, allowing the visualization of cancerous areas in real-time. Through this mechanism, [^18^F]FDG PET can detect and visually represent the specific glucose metabolism status in cancer tissues [39].

The use of [^18^F]FDG as an analog of glucose provides valuable insights into muscle activation. During cancer screening, exercise immediately before [^18^F]FDG injection may result in increased [^18^F]FDG uptake, necessitating exercise restriction prior to acquiring [^18^F]FDG uptake images for muscle imaging based on the same principle. Muscles are high-energy consumers, utilizing glucose for ATP (adenosine triphosphate) synthesis during muscle activity. [^18^F]FDG, which mirrors the physiological activity of glucose, is transported to the muscles via blood circulation. Within muscles, [^18^F]FDG undergoes oxidation and breakdown, similar to glucose, emitting radiation detectable by PET scanners, thus generating imaging that effectively visualizes muscle activity and energy consumption [26,27,39,40].

**Figure 1:**
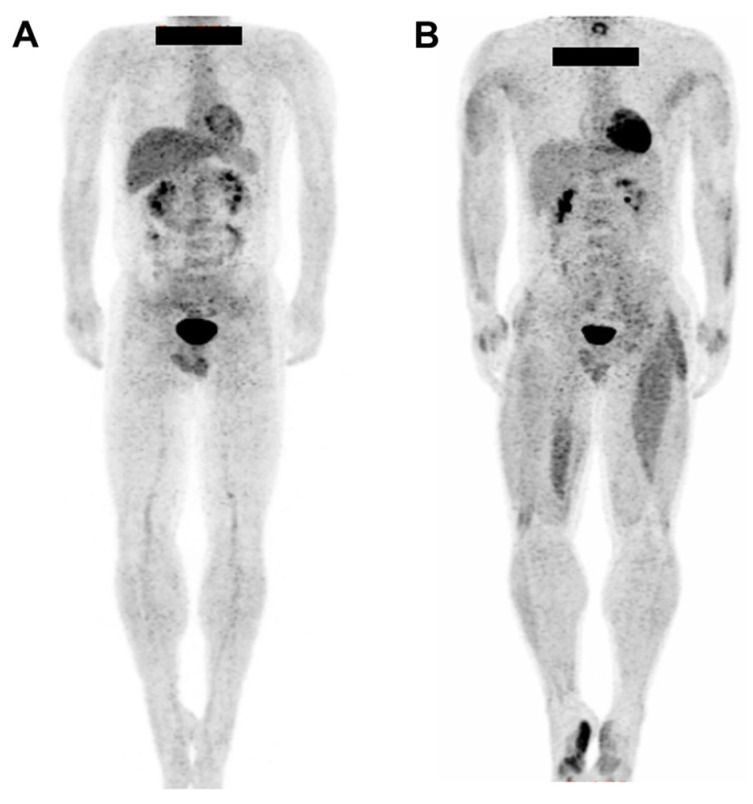
Representative images of whole-body positron emission tomography (PET) for the control (**A**) and baseball pitcher groups (**B**). Participants refrained from food and drink for a minimum of 6 h prior to the [^18^F]FDG PET evaluation and avoided intense physical activity for at least 1 day before the experiment. Prior to the injection of approximately 37 MBq of [^18^F]FDG, participants threw 40 baseballs, followed by 40 ball pinches. PET/CT images were obtained 50 min after injection, revealing a significant increase in glucose metabolism in the muscle groups within the utilized areas. Reproduced from [28], copyright © 2021, Journal of the International Society of Sports Nutrition.

In a study by Nakase et al., PET images of ten healthy men were obtained [26]. The group engaged in a progressively intensifying whole-body exercise program and exhibited induced glucose uptake in the abdominal rectus, gluteus, and small muscles, indicating the activation of these muscle areas. [^18^F]FDG PET can clearly identify the entire muscle-activated region (Figure 1). Utilizing PET advantages, several studies have successfully identified activated muscle areas during various exercises such as pinching, skiing, and pedaling [28,29,30,31,32,41].

**Figure 2:**
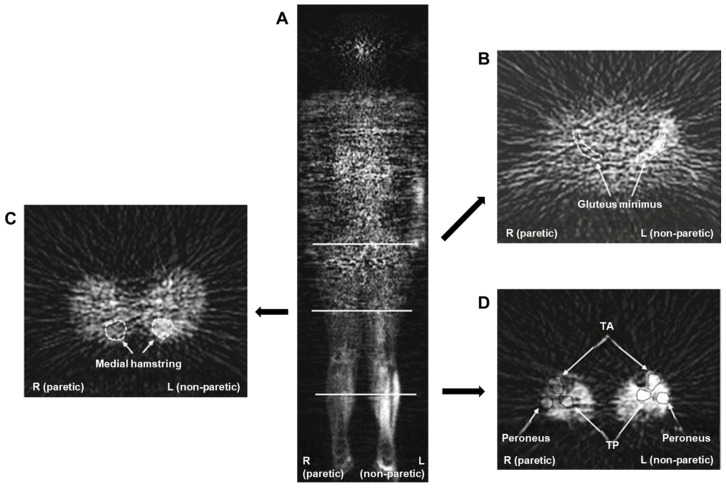
Representative images of whole-body positron emission tomography (PET) from a hemiparetic participant. (**A**) The coronal PET image (middle) is captured 6 cm in front of the subject’s posterior, depicting minimal [^18^F]fluoro-2-deoxy-D-glucose ([^18^F]FDG) uptake in the muscles of the affected (right) lower limb and elevated [^18^F]FDG uptake in the muscles of the unaffected (left) lower limb. This image provides an anterior–posterior view. (**B**) PET image in the axial plane of the pelvis showing reduced [^18^F]FDG uptake in the gluteus minimus muscle of the affected (right) lower limb. This image provides an inferior–superior view. (**C**) Axial PET image of the thigh showing increased [^18^F]FDG uptake in the medial hamstring muscle of the unaffected (left) lower limb. (**D**) Axial PET image of the lower leg showing reduced [^18^F]FDG uptake in the muscles of the affected (right) lower limb. TA, tibialis anterior muscle; TP, tibialis posterior muscle. Reproduced from [31], copyright © 2015, The Tohoku Journal of Experimental Medicine.

**Figure 3:**
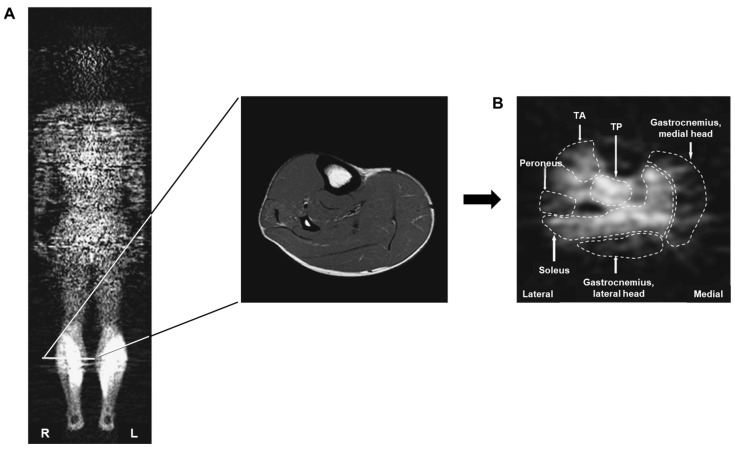
Representative images of whole-body positron emission tomography (PET) from a healthy participant. These images illustrate the delineation of regions of interest (ROIs) within each lower leg muscle on PET scans. (**A**) PET image was taken 6 cm in front of the subject’s posterior, providing an anterior–posterior perspective. The marked line indicates the axial magnetic resonance image used to identify the muscle. (**B**) An axial PET image (right lower leg) is used to define the ROI for each muscle, providing an inferior-to-superior perspective. TA, tibialis anterior muscle; TP, tibialis posterior muscle. Reproduced from [31], copyright © 2015, The Tohoku Journal of Experimental Medicine.

Furthermore, [^18^F]FDG PET is useful not only for muscle activation but also for confirming muscle inactivation. Oi et al. assessed the muscle status of patients with hemiplegic symptoms due to stroke using [^18^F]FDG PET to determine muscle activity [31]. The glucose uptake level (GUL) in the lower extremity muscle was significantly decreased in the paralyzed limb (Figure 2) compared to that in the non-paralyzed and healthy participants (Figure 3). The GUL of specific muscles in the paralyzed limb, including the tibialis anterior, tibialis posterior, nasopharyngeal, and obtuse muscles, was significantly decreased compared with the non-paralyzed side. Notably, the GUL of the gluteal, thigh, and lower limb muscles in the unparalyzed limbs of hemiplegic participants did not differ from those in healthy participants, except for the medial hamstring and tibialis posterior muscles.

These findings provide compelling examples for understanding the glucose uptake in muscles using [^18^F]FDG PET. This technique confirms the increased uptake in healthy muscles during exercise and provides insights into muscle volume. [^18^F]FDG is anticipated to be beneficial for assessing the effectiveness of exercise programs and distinguishing between healthy and damaged muscle tissue in patients. However, a distinct PET scanning protocol for cancer screening is necessary.

### 2.2. [^18^F]Fluorocholine ([^18^F]FCH)

Choline serves as a precursor of phosphatidylcholine, a vital component of the cell membrane, and acetylcholine, which is crucial in neural excitatory transmission. The choline analog [^18^F]fluorocholine ([^18^F]FCH) is a radiotracer for imaging prostate cancer, highlighting abnormal cell division and activation of choline kinase in prostate cancer tissues. This radiopharmaceutical identifies regions of increased metabolism in prostate cells, and its uptake is detected in real-time using a PET scanner, providing a visual confirmation of the precise location of cancerous tissues and abnormal metabolic activity [42].

Utilizing the properties of choline, [^18^F]FCH PET is a promising technique for skeletal muscle imaging. The hypothesis for the mechanism of action of the choline analog [^18^F]FCH in skeletal muscle is as follows: First, cholinergic neurons release acetylcholine stored at their terminals during muscle contraction, which is synthesized from choline and acetyl coenzyme A. Increased demand for acetylcholine synthesis during muscle contraction leads to elevated nearby choline uptake. Second, increased blood flow to activated muscles could contribute to the increased accumulation of [^18^F]FCH in the muscles [33,34,43]. The mechanism underlying [^18^F]FCH accumulation in healthy muscle tissues remains unclear and warrants further investigation, although evidence suggests its potential utility in muscle imaging.

In 2019, a case study was presented involving a 74-year-old patient with prostate disease [34]. PET scans revealed widespread and uniformly high [^18^F]FCH uptake, including the entire axial skeleton. Notably, the patient’s physical activity levels, including long walks, farming, and dance classes, likely contributed to the elevated [^18^F]FCH uptake, although it cannot be ruled out that it may reflect metastasis of prostate cancer, with increased local perfusion due to activity being the probable cause.

A study by Roef et al. is also noteworthy [35]. In their study, the experimental group was categorized into three subgroups: one engaged in high-intensity exercise, two without exercise (with activities outside of bed prohibited), and another with only light walking. The group performing high-intensity exercise using a 7.5 kg tool showed 202% higher uptake than those with restricted or no exercise.

**Figure 4:**
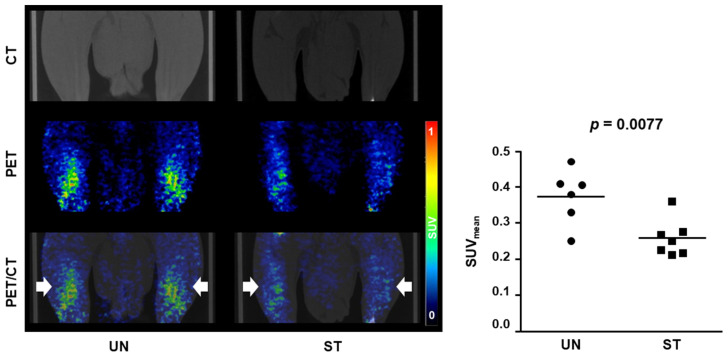
Comparison of [^18^F]FCH uptake by hindlimb skeletal muscle of control rats (UN) and rats in which muscle atrophy was induced by starvation (ST). Muscle atrophy model rats exhibited significantly lower [^18^F]FCH uptake than that of the control group. Choline uptake in atrophic muscle tissues was assessed using PET/CT with [^18^F]FCH. The volume of interest (VOI; white arrows) indicates both hindlimbs. The graph indicates the SUV_mean_ in the VOI of the control (UN, *n* = 6) and starvation groups (ST, *n* = 7). CT, computed tomography; PET, positron emission tomography; SUV_mean_, mean standardized uptake value; Reproduced from [13], copyright © 2022, Diagnostics.

Additionally, a preclinical study conducted in 2022 evaluated the utility of [^18^F]FCH for screening muscle atrophy [13]. This study confirmed that muscle atrophy was induced by starvation in rats and serum-starved L6 skeletal muscle cells, showing significantly lower uptake in the muscle atrophy model than in the control group (Figure 4). Anatomically, the soleus muscle exhibited significantly reduced mass, followed by the plantaris and gastrocnemius muscles. The muscle fiber area in the starvation group was significantly smaller than that in the control group. Histological analysis in this study confirmed the occurrence of starvation-induced muscle atrophy in rats through significant increases in MuRF-1 and Atrogin-1, which are indicators of muscle atrophy and protein degradation.

The uptake of choline and its analogs in muscles is undoubtedly associated with the level of muscle activation and the presence of disease. Although [^18^F]FCH has demonstrated efficacy as a diagnostic tool for muscle disorders at the preclinical level, further validation under diverse conditions is necessary, considering the genetic heterogeneity in rodents and humans. Additional research is required to elucidate the mechanism underlying [^18^F]FCH accumulation in healthy human muscles.

### 2.3. [^11^C]Acetate

The acetate analog [^11^C]acetate is a radiopharmaceutical used in prostate cancer staging and cardiac research, particularly in investigating parameters such as myocardial oxygen consumption and blood flow [36]. Acetate is converted to acetyl coenzyme A within cells, contributing to energy production in the mitochondria. To generate adenosine triphosphate, which is required for cellular energy production, cells must undergo a metabolic pathway known as oxidative phosphorylation. Acetate, a free fatty acid, is promptly converted into acetyl coenzyme A and enters the tricarboxylic acid (TCA) cycle. This enables the measurement of the TCA cycle flux and the production of reducing equivalents. Because this process is associated with oxygen consumption, utilizing [^11^C]acetate allows the estimation of flux through the TCA cycle by measuring oxygen consumption [44]. Moreover, the uptake of [^11^C]acetate in the muscle tissues appears to reflect muscle metabolism [36,44].

In a 2015 study by Trombella et al., electrically induced contractions in one hind limb muscle were compared with those in the unstimulated contralateral limb [44]. In the one-leg knee extensor exercise model, the stimulated limb exhibited uptake of [^11^C]acetate. However, the SUV values (in static PET) struggled to effectively compare exercised and resting limbs. Dynamic PET imaging and kinetic analyses provide a clear understanding of these differences.

**Figure 5:**
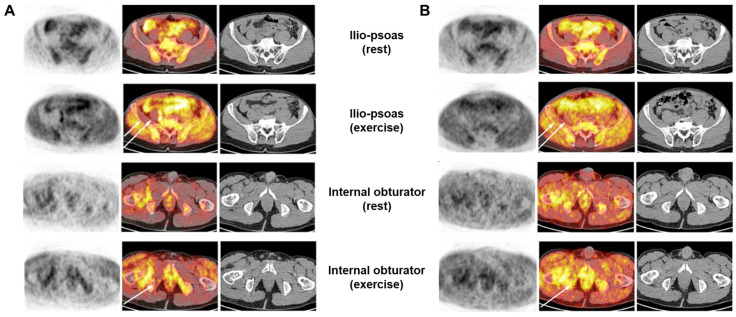
PET/CT with [^11^C]acetate was conducted at three weeks (**A**) and three months (**B**) after right hip arthroplasty using the posterior “Kocher Langerhans” approach. The images depict the iliopsoas and obturator muscles, with the upper and lower rows displaying resting and after-exercise PET, respectively. In each row, from left to right, the images represent PET alone, PET/CT superposition, and low-dose CT alone. The notable surge in [^11^C]acetate uptake during exercise was similarly observed on the unaffected side of the body in the iliac, psoas, and internal obturator muscles. Conversely, on the operated side, these muscles (indicated by arrows) exhibited similar activity levels during rest and exercise at the 3-week mark. The postsurgical [^11^C]acetate PET hyperactivity extending into the external obturator muscle on the right side, directly related to the surgical approach, remained stable at rest and exercise. The iliopsoas and obturator muscles affected by surgery ((**B**), indicated by arrows) show almost complete recovery. [^11^C]acetate uptake from surgical sites increased with exercise, similar to [^11^C]acetate uptake from healthy body sites. CT, computed tomography; PET, positron emission tomography. Reproduced from [36], copyright © 2011 Molecular Imaging and Biology.

In related clinical studies, there have been cases where the recovery progress of damaged skeletal muscles after surgery was assessed using [^11^C]acetate [36]. Patients (*n* = 2) who underwent hip replacement surgery underwent scans three and twelve weeks postoperatively while performing rehabilitation exercises. Scans were conducted during resting and exercise periods in each set (Figure 5). Initially, when comparing exercise and rest, the uptake of [^11^C]acetate was higher during exercise than during rest. At three weeks postoperatively, four muscles related to the procedure lacked exercise participation, suggesting potential muscle function impairment. This absence was temporary, with regained exercise participation observed three months postoperatively. This study indicates the potential of [^11^C]acetate PET as a non-invasive tool for assessing muscle function and detecting occasional impairments after arthroplasty.

Understanding the mechanism by which [^11^C]acetate accumulates in the skeletal muscles and determining an appropriate PET scanning method seem to be necessary. Although further research on [^11^C]acetate is required, it holds promise for measuring muscle metabolism and obtaining information on muscle mass.

### 2.4. [^15^O]Water ([^15^O]H_2_O)

Blood flow is crucial in muscle physiology and is closely associated with muscle mass and activation levels. A sufficient blood supply ensures the delivery of oxygen, nutrients, and energy required for muscle function. Enhanced blood flow supports muscle growth, functionality, energy production, and waste removal during activities, contributing to overall muscle health and performance. Enhancing muscle strength is closely linked to the promotion of arterial function and the proliferation of capillaries through increased blood flow to the muscles. Utilizing [^15^O]H_2_O PET, changes in muscle blood flow can be accurately quantified, as it has been successfully applied to assess regional blood flow in vital organs such as the brain and heart. Previous studies used [^15^O]H_2_O PET to evaluate muscle blood flow [37,38].

Ruotsalainen et al. assessed muscle blood flow using autoradiographic and steady-state methods and demonstrated its efficacy for measuring local blood flow by comparing it with [^15^O]H_2_O PET [45]. These methods facilitate the accurate measurement of blood flow in skeletal muscles, allowing for the visualization of blood flow distribution and metabolite exchange within muscle tissues.

## 3. Discussion

Our objective was to assess the efficacy of muscle PET technology for muscle imaging based on case studies and articles. Muscle PET imaging has emerged as a valuable diagnostic tool for detecting muscle atrophy and screening for hypertrophy. Historically, dual-energy X-ray absorptiometry, CT, MRI, and ultrasonography have been used to measure the relationship between muscle mass and muscle atrophy. Although these methods reliably quantify muscle mass, there is no consensus regarding which indicators of muscle quality possess the most prognostic value. For instance, CT enables consistent quantitative tissue measurements using specific datasets. However, these anatomical imaging modalities cannot evaluate the molecular activity of skeletal muscles [38,46]. In contrast, PET is sensitive to glucose and fatty acid metabolism, as well as blood flow, allowing the assessment of regions of interest and tissue activation levels. Additionally, PET offers the advantage of providing systemic information encompassing the entire body rather than local tissues. These attributes render muscle PET imaging valuable for assessing skeletal muscles across conditions ranging from diabetes- and cancer-related dysfunction to exercise physiology.

Despite its potential benefits, our literature review has revealed several limitations of muscle PET imaging. One significant gap is the absence of a standardized imaging protocol for muscle PET imaging, which is noteworthy. Such a protocol may include stages, such as light exercise, during the uptake phase following radiopharmaceutical administration. Patients should be able to perform these protocol stages effectively to achieve optimal diagnostic outcomes, emphasizing the need for protocol optimization. Furthermore, limited accessibility poses a challenge, primarily owing to the cost factors associated with PET imaging, which involve the use of radiopharmaceuticals, resulting in relatively high expenses and low-level radiation exposure. Although radiation exposure during individual scans remains within safe limits, cumulative exposure from repeated imaging may pose health risks. Hence, careful consideration of these limitations is warranted when using muscle PET.

Furthermore, while not covered in this review, a few studies on skeletal muscle imaging using radiopharmaceuticals such as 3’-[^18^F]fluoro-3’-deoxythymidine ([^18^F]FLT), [^18^F]fluoromisonidazole ([^18^F]FMISO), [^64^Cu]diacetyl-bis(*N*^4^-methylthiosemicarbazone) ([^64^Cu]Cu-ATSM), and ^68^Ga-fibroblast activation protein inhibitor ([^68^Ga]Ga-FAPI-04), which are primarily used for tumor imaging, have been reported [47,48,49,50,51]. These studies presented preliminary findings, suggesting the potential value of exploring more specific radiopharmaceuticals to assess skeletal muscle injury and recovery.

## 4. Conclusions

As a functional imaging technique, PET scanning provides crucial information about the target area, depending on the radiopharmaceutical employed. Although extensively utilized in oncology due to its efficacy, PET imaging has the potential for applications in muscle assessment. It enables volumetric and uptake analyses, providing insights into the skeletal muscle areas that absorb radioisotope-labeled ligands. Radiopharmaceuticals such as [^18^F]FDG, [^18^F]FCH, [^11^C]acetate, and [^15^O]H_2_O demonstrate effectiveness in muscle imaging despite certain limitations. Nonetheless, PET imaging using biomarker-specific targeting of radiopharmaceuticals remains a valuable tool for muscle examination and diagnosis.

## Figures and Tables

**Table 1:** Features of the original articles reviewed.

Radiotracer	Subjects	Procedure	Main Results	Ref.
[^18^F]FDG	Healthy volunteers (each group *n* = 5)	Scans were conducted following an injury-prevention exercise program.	The exercise group demonstrated higher [^18^F]FDG accumulation than that of the control group in the abdominal rectus, gluteus medius, and minimus muscles.	[26]
	Patients (*n* = 1164)	Whole-body [^18^F]FDG PET scans were performed.	Of 1164 patients, 146 exhibited excessively increased muscle uptake, corresponding to technologists’ observations of muscle activity during the uptake phase or prior to [^18^F]FDG injection.	[27]
	Skilled pitchers and healthy volunteers (each group *n* = 10)	Participants threw 40 baseballs before receiving an injection of [^18^F]FDG, followed by 40 pitches. PET-CT images were acquired 50 min after the injection of [^18^F]FDG.	Significant increases in glucose metabolism were observed in the muscle groups of the fingers and toes on the throwing and non-throwing sides. Symmetric increases in glucose metabolism were also noted in the thigh muscles.	[28]
	Healthy volunteers (*n* = 8)	[^18^F]FDG was injected during exercise, followed by a whole-body PET scan conducted after the exercise.	[^18^F]FDG uptake did not increase in the upper body; however, increased uptake was observed in the lower body muscles, including the knee flexors and extraterrestrial and abdominal muscles.	[29]
	Healthy volunteers (*n* = 20)	[^18^F]FDG was injected 10 min after the start of exercise or after 20 min of rest. Whole-body PET scan was conducted after the exercise or rest periods.	The exercise group exhibited significantly greater uptake than that of the control group in the iliacus muscle and muscles of the anterior part of the thigh.	[30]
	Hemiparetic patients (*n* = 8)	Whole-body [^18^F]FDG PET scans were conducted after walking.	The [^18^F]FDG uptake levels in the flounder, foreground, posterior tibia, and obtuse muscles were significantly reduced in the paralyzed muscles of hemiplegic patients compared to non-paralyzed muscles of healthy participants.	[31]
	Sprague-Dawley rats (each group *n* = 4)	Rats underwent 3 days of fasting followed by [^18^F]FDG PET imaging scans.	Rats induced with muscle atrophy exhibited significantly reduced [^18^F]FDG uptake compared to that of the control group.	[32]
[^18^F]FCH	Patient with a history of prostate neoplasm	Patients were scanned 5, 45, and 120 min after [^18^F]FCH injection; post-5 min-Pelvic image scans were conducted, while whole-body scans were performed at 45 and 120 min postinjection.	The scans revealed heterogeneous [^18^F]FCH uptake throughout the body, attributed to intense physical activity.	[33]
	Sprague-Dawley rats (each group *n* = 4)	Rats were starved for 48 h and then scanned for [^18^F]FCH PET imaging.	[^18^F]FCH uptake of hind leg muscle was significantly lower in the muscle atrophy-induced model than in the control group.	[13]
	Patients (*n* = 10)	Three groups with different [^18^F]FCH injection and absorption conditions were compared. Group 1: Strict (bed) rest Group 2: Allowed to walk Group 3: Performed strenuous single-arm exercise	Strenuous exercise significantly increases muscle absorption. Strict bed rest did not show significantly lower muscle absorption compared to walking for a short distance.	[34]
[^11^C]Acetate	Patients undergoing hip arthroplasty (*n* = 2)	Investigated 3 and 12 weeks after hip arthroplasty with the muscle at rest and during exercise.	Increased [^11^C]acetate uptake of muscles indicated the degree of functional recovery after hip arthroplasty.	[35]
	Wistar rats (*n* = 8)	Both legs of the one-leg knee-extensor excise model were compared.	The rate at which the exercised leg absorbed the [^11^C]acetate was faster than that of the non-exercised leg.	[36]
[^15^O]H_2_O	Healthy volunteers (*n* = 20)	Quantitation of blood flow in the thigh muscles was measured using autoradiography and [^15^O]H_2_O PET (steady state flow).	Although blood flow could be obtained through [^15^O]H_2_O PET, it was less efficient than autoradiography.	[37]
	Control group (*n* = 5) and normoglycemic hyperinsulinemic (*n* = 7)	After injecting bradykinin into the femoral artery, blood flow in both femurs was measured using [^15^O]H_2_O.	Blood flow in the skeletal muscles could be measured.	[38]

PET, positron emission tomography; CT, computed tomography; [^18^F]FDG, [^18^F]fluoro-2-deoxy-D-glucose; [^18^F]FCH, [^18^F]fluorocholine; [^15^O]H_2_O, [^15^O]water.
